# UV-B radiation enhances isoflavone accumulation and antioxidant capacity of soybean calluses

**DOI:** 10.3389/fnut.2023.1139698

**Published:** 2023-03-30

**Authors:** Mian Wang, Guannan Liu, Tianwei Guo, Chong Xie, Pei Wang, Runqiang Yang

**Affiliations:** College of Food Science and Technology, Nanjing Agricultural University, Nanjing, Jiangsu, China

**Keywords:** UV-B radiation, isoflavone, soybean callus, antioxidant capacity, physiological metabolism

## Abstract

Isoflavones are a class of flavonoids that belong to a large family of polyphenols and synthesized predominantly in legume, and they play important roles including acting as antioxidant, preventing osteoporosis, reducing the risk of atherosclerosis, and protecting against cardiovascular disease. This study focused on the accumulation and synthetic metabolism of isoflavone in soybean hypocotyl and cotyledon calluses under UV-B radiation. The results showed that UV-B radiation significantly up-regulated the gene expression of phenylalanine ammonia lyase (PAL), cinnamate-4-hydroxylase (C4H), 4-coumarate-CoA ligase (4CL), chalcone ketone synthase (CHS), chalcone isomerase (CHI), and isoflavone synthase (IFS), and enhanced their activity in soybean hypocotyl and cotyledon calluses. As a result, isoflavones content increased by 21.23 and 21.75% in soybean hypocotyl and cotyledon calluses, respectively. Among the isoflavones produced, malonyldaidzin was the dominant one in hypocotyl callus, while malonylglycitin and daidzein were the main isoflavones in cotyledon calluses. This study revealed that UV-B radiation induced isoflavone accumulation in soybean calluses, which could be an efficient strategy to improve the nutritional value of food and produce high levels of bioactive secondary metabolites.

## 1. Introduction

Soybeans (*Glycine max* L.) are a major economic crop that provide high-quality protein and other bioactive metabolites. Isoflavones are the main secondary metabolites in soybean, and they are related to plant defense responses and health-related benefits for human (1). Isoflavones are synthesized from phenylalanine and can be divided into four groups (12 different monomers): aglycones (include daidzein, genistein and glycitein), glycosides (include daidzin, genistin, and glycitin), malonylglycosides (include malonyldaidzin, malonylgenistin, and malonylglycitin), and acetylglycosides (include acetyldaidzin, acetylgenistin, acetylglycitin) (2). Among them, glycosides and malonylglycosides account for 97−98% of total isoflavone in soybean (2), and acetylglycosides are present in very low quantities. The content of isoflavone in soybean seeds is typically 0.2−1.6 mg/g dry weight (DW) (3).

Abiotic stress is usually used to enrich bioactive metabolites in plants. Ultraviolet B (UV-B, 280−315 nm) radiation can cause physiological and biochemical changes in plants by damaging photosynthesis tissues and altering DNA structure of cells. It up-regulates key genes expression involved in the phenylpropanoid metabolic pathway, leading to the increased enzymes activity and enhanced synthesis of isoflavone (4). Plant calluses grows rapidly, and has the ability to synthesize isoflavone. Calluses can be easily cultivated in controlled conditions and are not susceptible to external environment. Therefore the tissue culture technique is often used to promote the synthesis of secondary metabolites, such as isoflavone, paclitaxel, alkaloids, carotene, and glucosinolate (5). At present, the combination of tissue culture technique and UV-B radiation treatment has been extensively used to enrich biological metabolites to improve the nutritional value of food. Mohajer et al. (6) found that the survival rate of sainfoin (*Onobrychis viciifolia* Scop.) seeds decreased significantly under UV-B radiation, but flavonoids and phenolics content in their calluses increased. The effect of UV-B radiation on phenolic compounds, total flavonoids content and antioxidant capacity of *in vitro* grown *Echium orientale* L. shoots and calli was investigated (7). Phenolics and flavonoids content, especially rosmarinic acid and quercetin, increased in callus extracts of *Echium orientale* L. which were harvested after 1 week of UV-B treatment, resulting in the enhancement of their antioxidant activity (7). Besides, tempisque (*Sideroxylon capiri* Pittier) is classified as an endangered species and has a high level of phenolics and flavonoids in its leaves. Phenolics and flavonoids content increased in the fourth week with 4 h of UV-B exposure per day, and the highest concentrations of quercetin (230 μg/g DW), kaempferol (235 μg/g DW) and gallic acid (240 μg/g DW) were found in callus obtained from leaves explants (8). Mata-Ramírez et al. (9) evaluated the quantification and bioactivity of isoflavone in soybean callus submitted to UV-light stresses. It was found that UVC-light stress increased genistein-O-glucoside and genistein-O-glucosyl-malonate to 122 and 196% in soybean callus, respectively, resulting in the enhancement of anti-inflammatory and antioxidant activity in calluses. Therefore, the combination of tissue culture technique and UV-B radiation treatment is one of the effective ways to obtain bioactive compounds. This method is not affected by climate fluctuations, and reduce dependence on land and other natural resources. Besides, it ensures the consistency between production batch under controlled conditions.

Previous researches have explored the use of tissue culture technique combined with UV-B radiation to obtain biological metabolites. However, there are scarce reports about the effects of UV-B radiation on the accumulation of isoflavones in soybean calluses, as well as the composition and content of isoflavones in different soybeans calluses. The vitality of key enzymes in different soybean calluses is also unclear. Thus, the aim of this study was to investigate the formation of soybean hypocotyl and cotyledon calluses, assess the effects of UV-B radiation on the activity and gene expression of key enzymes in isoflavone synthesis, and analyze the composition and content of isoflavone, as well as their antioxidant activity. This study can help to improve the productivity of secondary metabolites with biological activity, and provide an efficient strategy for improving the nutrition quality of soybean-based processed food.

## 2. Materials and methods

### 2.1. Chemicals

Soybean seeds (*Glycine max* L., cultivar Dongnong, 2021) were provided by Jiangsu Academy of Agricultural Sciences, China, and stored at −20°C. This variety was selected from several soybeans according to the content of isoflavones. All reagents and solvents were analytical or higher grade and purchased from Sigma (Sigma-Aldrich GmbH, Shanghai, China), Sinopharm (Sinopharm Chemical Reagent GmbH, Shanghai, China) and Maclean (Maclean Biotechnology GmbH, Shanghai, China), including Murashige and Skoog (MS) basal medium, 1/2 MS medium (without sugar and agar), 2, 4-dichlorophenoxyacetic acid (2, 4-D), 6-Benzylaminopurine (6-BA), sucrose, agar, acetonitrile, 1,1-diphenyl-2-trinitrophenyl hydrazine (DPPH), 2,2-diazo-di (3-ethyl-benzothiazole-6-sulfonic acid) diammonium salt (ABTS) and 6-hydroxy-2,5,7,8-tetramethylchromone-2-carboxylic acid (Trolox), methanol (AR), ethyl acetate, cinnamic acid, coenzyme A (CoA), coumaric acid, L-phenylalanine, rutin, gallic acid, etc.

### 2.2. Plant material and surface sterilization

Soybeans were exposed to sodium hypochlorite (1%) for 15 min and manual oscillation, then rinsed in distilled water. Seeds were dipped into ethanol 75% (v/v) solution for 5 min, and washed with distilled water for 4−5 times. Finally, they were submerged in anhydrous ethanol for 30 s. After that, seeds were inoculated in the 1/2 Murashige and Skoog salt mixture (1/2 MS) media (with 3% sucrose + 0.7% agar, Ph 5.8) at 25°C for 2 days. Then, they were cultivated in the Murashige and Skoog salt mixture (MS) media (with 3% sucrose + 0.7% agar, pH 5.8) for 4 days (16 h/8 h light/dark). The 6-day-old sterile soybean sprouts were used to obtain hypocotyl and cotyledon.

### 2.3. Elicitation of soybean hypocotyl and cotyledon calluses

Optimization of hormone concentration in induced medium: Samples (hypocotyls or cotyledons) were separated from the 6-day-old sterile soybean sprouts, and cut into slices with a thickness of about 2 mm, flat side up, and then placed on Murashige and Skoog salt mixture (MS) media (with 3% sucrose + 0.7% agar, pH5.8). Explants were cultured at 25°C in light (16/8 h light/dark) and subcultured on the same medium every 7 days until the callus formation was observed (28 days). Five calluses were placed on per petri dish (90 × 20 mm). The hormones in the MS media were 2, 4-D (1 and 2 mg/L) and 6-BA (0.5 mg/L). The concentration and ratio of hormones were determined based on the growth state and total isoflavone content of callus (Supplementary Table 2).

Determination of UV-B radiation intensity: Calluses were cultivated for 28 days and then were treated with UV-B (Intensities: 20, 40 and 80 μW/cm^2^, respectively) (Beijing Electric Power Source Research Institute, Beijing, China) at 25°C for 2 h, respectively. After that, calluses were kept in the same incubation conditions for 48 h in the dark. Isoflavone content was measured to determine the optimal UV-B radiation intensity (Supplementary Table 3). Soybean callus without UVB-light exposure was considered as the control.

Determination of re-incubation time: Soybean calluses were cultivated for 28 days and then irradiated by 40 μW/cm^2^ UV-B for 2 h. Then, calluses were kept in the same incubation conditions for 0, 6, 12, 24, 36, 48 h in the dark. Isoflavone content was measured to determine the optimal re-incubation time (Supplementary Table 4).

#### 2.3.1. Cultivation condition and treatments

Hypocotyls and cotyledons were separated from the 6-day-old sterile soybean sprouts, and cut into slices with a thickness of about 2 mm, flat side up, then cultured on MS media with 3% sucrose, 0.7% agar supplemented with 1 mg/L 2, 4-D and 0.5 mg/L 6-BA (pH 5.8) at 25°C in light (16 h/8 h light/dark) for 28 days. The medium was changed every 7 days. Calluses were irradiated by 40 μW/cm^2^ UV-B for 2 h, then re-incubated for 12 h in the dark (T). Soybean calluses without UVB-light exposure were considered as the control (CK). Afterward, some of calluses were frozen-dried and milled to powder in an electric mill (A11 Basic Analytical Mill, IKA, Guangzhou, Guangdong, China), stored at −20°C for further measurements. The remaining calluses were frozen with liquid nitrogen and stored at −80°C until analysis.

### 2.4. Determination of malondialdehyde content and electrolyte leakage

Malondialdehyde (MDA) content of soybean calluses was determined as previously reported by Wang et al. (10) with some modifications. The absorbance values of samples were measured at 450 nm, 532 nm, and 600 nm, respectively. The MDA content was expressed as nmol/g fresh weight (FW).

To determine the electrolyte leakage of soybean calluses, 1.0 g of the samples were cut into small pieces (3 × 3 × 3 mm^3^), and immersed in 30 mL of distilled water, then shaken at 25°C for 1 h. The conductivity was measured as EC_1_. The reaction liquid was boiled for 10 min to inactivate the plant tissue. After cooling, the volume was adjusted to 30 mL, and the boiling conductivity was measured as EC_2_. Electrolyte permeability (%) = (EC_1_/EC_2_) × 100%.

### 2.5. Growth parameters

The biomass of soybean calluses was determined by drying at 50°C until reaching a constant weight. The dry weight was measured every 3 days, and then the growth curve of hypocotyl and cotyledon calluses were plotted, respectively. There were three calluses in each dish.

### 2.6. Morphological observation

Sample materials and dehydration: Soybean calluses were cut into 3 × 3 × 3 mm^3^ small pieces and immersed in FAA solution (70% ethanol: ice acetic acid: 38% formaldehyde = 18: 1: 1) for 48 h. Then, samples were dehydrated with 70% ethanol for 30 min, 85% ethanol for 30 min, 95% ethanol for 30 min, and ethanol for 20 min in order.

Waxing, embedding and producing: Samples were immersed in wax and kept at 42°C for 24 h, then heated at 58°C to remove dimethylbenzene. The wax was changed every 6 h. After that, samples were embedded and produced according to the method of paraffin slices (11). The thickness of each slice was 10 μm.

Dewaxing and dyeing: The slices were dewaxed with dystershopine for 40 min, ethanol for 10 min, 75% alcohol for 5 min, and then washed with distilled water. The slices were dyed in the Safranin O solution for 2 h, washed with distilled water, and then immersed in 50, 70, and 80% of alcohol for 5 s in order. After that, the slices were immersed in fast green stain for 30 s, dehydrated with ethanol, and finally immersed in dimethylbenzene for 5 min, sealed and fixed with optics resin. The cell morphological structure of soybean calluses was observed in optical microscope (Eclipse Ci-L, Nikon, Chiyoda, Tokyo, Japan).

### 2.7. Quantification of total flavonoids content

The 0.05 g of frozen-dried sample was shaken at 25°C for 1 h with 80% methanol, centrifuged at 10,000 × *g* for 15 min at 4°C, and then the supernatant was collected. Three replicates were conducted and the supernatant was filtered. The filtrates were evaporated and concentrated at 40°C. The residue was dissolved with 50% methanol to 5 mL as free phenolic solution (FPS), and finally stored at −20°C until analysis.

After the extraction of FPS, the residue was shaken with 2 mol/L of NaOH at 25°C for 4 h, and pH was adjusted to 1.6−1.8. The sample was extracted with ethyl acetate for 15 min at room temperature, then centrifuged at 10,000 × *g* for 15 min at 4°C. The ethyl acetate layer was collected. Three replicates were conducted, and the combined supernatant was evaporated to dry at 40°C, and dissolved with 50% methanol to 5 mL, as bound phenolic solution (BPS), finally stored at −20°C until analysis.

Determination of total flavonoid content was based on the method described by Miliauskas et al. (12) using soybean calluses as experimental samples and rutin as the standard. Results were expressed as milligram (mg) of rutin equivalents (RE) per 100 g of DW.

### 2.8. Determination of antioxidant capacity

2,2-diazo-di (3-ethyl-benzothiazole-6-sulfonic acid) diammonium salt and 1,1-diphenyl-2-trinitrophenyl hydrazine radical scavenging activities of soybean calluses were measured as described by Chen et al. (13) using FPS and BPS as samples and Trolox as the standard. They were expressed as micromole of Trolox equivalents per gram (g) of DW.

### 2.9. Identification and quantification of isoflavone by high-performance liquid chromatography

Soybean callus extract preparation: 100 mg of callus was weighed and mixed with 5 mL 80% (v/v) methanol, and then sonicated for 15 min at 40°C. After sonication, samples were centrifuged at 10,000 × *g* for 10 min at 4°C, and the supernatants were filtered with a 0.45 μm membrane filter for Agilent 1200 HPLC system (Agilent Technologies Co., Ltd., Santa Clara, CA, USA). Typical HPLC chromatograms of nine isoflavone monomers in soybean hypocotyl and cotyledon calluses were shown in Supplementary Figure 2. The mixed standard concentration selected in this study was 3.2, 6.4, 12.8, 25.6, and 51.2 μg/mL.

A reversed phase column (Ultimate AQ-C18, 4.6 × 250 mm, 5 μm particle size) was used. Mobile phase A consisted of 0.1% acetic acid in water, and mobile phase B consisted of 0.1% acetic acid in acetonitrile. A 52 min gradient was programmed as follows: 0−50 min, 13−35% B; 50−51 min, 35−13% B; 51−52 min, 13% B. The column temperature maintained at 35°C. The injection volume was 20 μL and the flow rate was 1.0 mL/min. The measurement wavelength was 260 nm.

### 2.10. Determination of key enzymes activity in isoflavone synthesis

Determination of phenylalanine ammonia lyase (PAL), cinnamate-4-hydroxylase (C4H) and 4-coumarate-CoA ligase (4CL) activity was based on the method of Ma et al. (14) with slight modification. The activity was expressed as U/g FW.

The activity of chalcone ketone synthase (CHS), chalcone isomerase (CHI), and isoflavone synthase (IFS) was determined using CHS ELISA Kit (MBE21206), CHI ELISA Kit (MBE21201), and IFS ELISA Kit (MBE21199), respectively. The kits were all from Nanjing Jiancheng Institute of Biological Engineering (Nanjing, China). The 1.0 g of soybean calluses were full ground with 5 ml 9% physiological saline, centrifuged at 3,500 × *g* for 15 min at 4°C. The supernatant was collected, and analyzed according to the instructions. The activity was expressed as IU/g FW.

### 2.11. Quantitative RT-PCR

Total RNA isolation and real-time PCR were conducted according to Wang et al. (15) the reference gene was *En- longation Factor 1b* (*EF1b*). The primers for real-time PCR analysis of *PAL*, *C4H*, *4CL*, *CHS*, *CHI*, *IFS*, and *EF1b* were designed using Primer 5.0 primer design software according to primers published on NCBI, synthesized by GenScript Biotechnology Co., Ltd. (Nanjing, China). The detected genes and sequences of primers were listed in Supplementary Table 1.

### 2.12. Statistical analysis

Experimental data were expressed as mean ± standard deviation (SD) with three replications (*n* ≥ 3). SPSS 18.0 (SPSS Inc., Chicago, IL, USA) was applied for significant difference tests. Data were analyzed by Duncan’s multiple-range tests at *p* < 0.05.

## 3. Results

### 3.1. Physiological metabolism of soybean calluses under UV-B radiation

**FIGURE 1:**
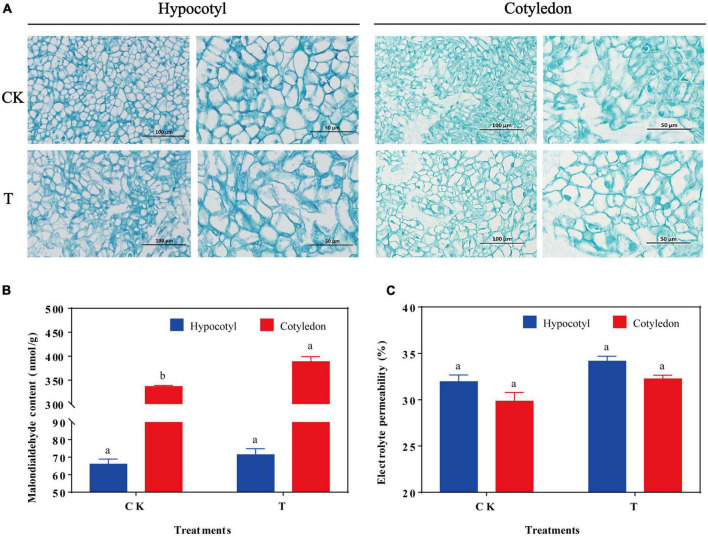
Effects of UV-B on cell structure **(A)**, MDA content **(B)**, and electrolyte leakage **(C)** of soybean hypocotyl and cotyledon calluses. The lower case letters indicated significant difference at *p* < 0.05 between treatments, *T*-test was used for data analysis. The data were presented as mean ± SD, *n* = 3.

The soybean hypocotyl and cotyledon calluses were cultured for 28 days (Supplementary Figures 1A, B), and their biomass increased significantly from days 3−18 (Supplementary Figures 1C, D). When exposed to UV-B radiation, the cells were swollen and occasionally ruptured in soybean calluses (Figure 1A). Besides, MDA content in soybean cotyledon calluses significantly increased by 15.74% compared with the control (CK), and was higher than the content of hypocotyl callus (Figures 1B, C). However, there was no significant difference on the electrolyte permeability in soybean hypocotyl and cotyledon calluses.

### 3.2. Total flavonoids content and antioxidant capacity of soybean calluses

**FIGURE 2:**
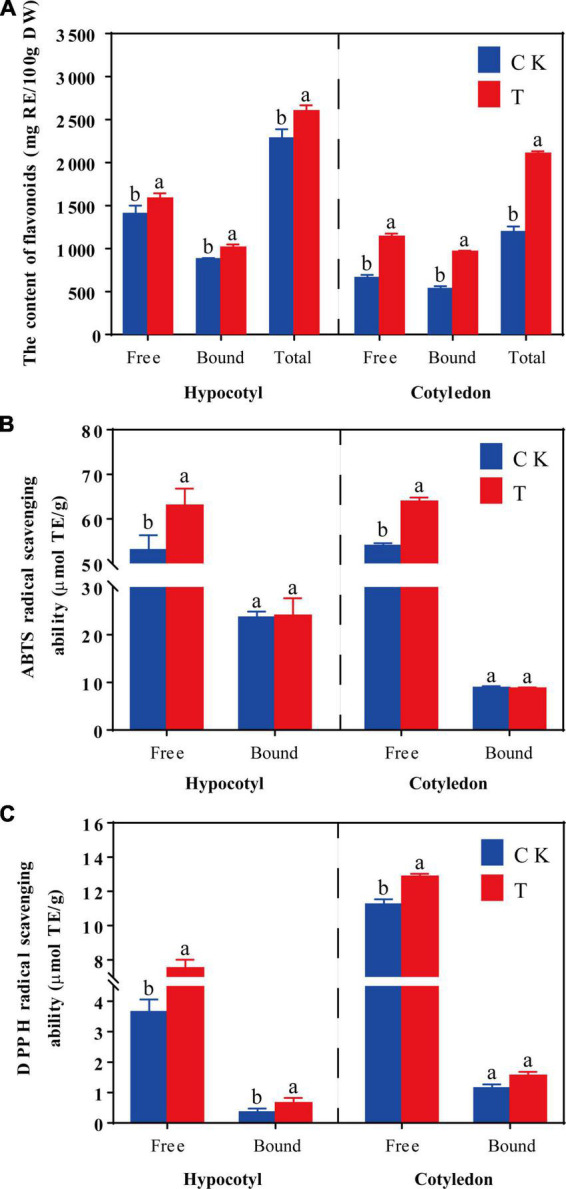
Effects of UV-B on the flavonoids content **(A)**, ABTS **(B),** and DPPH **(C)** radical scavenging activity of soybean hypocotyl and cotyledon calluses. The lower case letters indicated significant difference at *p* < 0.05 between treatments, and *T*-test was used for data analysis. The data were presented as mean ± SD, *n* = 3.

Total flavonoids content and the antioxidant activity of soybean calluses are shown in Figure 2. The content of free, bound and total flavonoids content increased by 12.81, 15.47, and 13.83% in soybean hypocotyl calluses under UV-B radiation, respectively, compared with the control (Figure 2A). And the content of free, bound and total flavonoids content significantly increased by 72.73, 81.25, and 76.54% in soybean cotyledon calluses under UV-B radiation, respectively. Results showed that total flavonoid content was higher in hypocotyl calluses, but cotyledon calluses were more sensitive to UV-B radiation to accumulate isoflavone.

The effects of UV-B on the *vitro* antioxidant properties of soybean calluses were represented by ABTS (B) and DPPH (C) radical scavenging ability (Figures 2B, C). The antioxidant capacity of phenolics increased significantly (*p* < 0.05) under UV-B radiation, especially free phenolics. As shown in Figure 2B, ABTS free radical scavenging ability of free phenolics increased by 18.85 and 18.32% in soybean hypocotyl and cotyledon calluses under UV-B radiation, respectively, compared with the control (Figure 2B). There was no significant difference on the ABTS free radical scavenging ability of bound phenolics in soybean calluses. The DPPH free radical scavenging capacity of free phenolics significantly increased by 106.16 and 14.49% in soybean hypocotyl and cotyledon calluses under UV-B radiation, respectively, compared with the control (Figure 2C). And the DPPH free radical scavenging capacity of bound phenolics improved only in soybean hypocotyl callus, increased by 83.33% compared with the control. Indicating that UV-B radiation could significantly promote the synthesis of total flavonoids, and improve cellular antioxidant activity in soybean calluses.

### 3.3. Isoflavone composition and content in soybean calluses

The content of individual isoflavone in soybean hypocotyl and cotyledon calluses under UV-B radiation is shown in Table 1. Nine major isoflavones were detected, including three groups: aglycones (daidzein, genistein, and glycitein), glycosides (daidzin, genistin, and glycitin), and malonylglycosides (malonyldaidzin, malonylgenistin, and malonylglycitin). Compared with the control, isoflavone content increased by 21.23 and 21.75% in soybean hypocotyl and cotyledon calluses under UV-B radiation, respectively. The contents of individual isoflavone in different soybean calluses varied. In soybean hypocotyl callus, the content of daidzin, genistin, malonyldaidzin and malonylglycitin increased, but malonylgenistin content decreased compared with the control. Glycitin, daidzein, glycitein and genistein were not detected. In soybean cotyledon callus, four kinds of individual isoflavone including malonyldaidzin, malonylglycitin, daidzein and glycitein showed the relatively higher content compared with the control. And there was no significant difference in the content of glycitin and genistin. Daidzin, malonylgenistin and genistein were not detected.

**TABLE 1:** The content of individual isoflavone (μg/g) in soybean calluses under UV-B radiation.

Isoflavone	Hypocotyl		Cotyledon	
	**CK**	**UV-B**	**CK**	**UV-B**
Daidzin	74.81 ± 0.88^b^	161.89 ± 9.15^a^	ND	ND
Glycitin	ND	ND	201.86 ± 14.51^a^	188.45 ± 10.55^a^
Genistin	82.02 ± 1.87^b^	102.01 ± 1.42^a^	135.78 ± 4.42^a^	146.93 ± 4.80^a^
Malonyldaidzin	1,696.05 ± 33.72^b^	2,400.35 ± 107.78^a^	119.57 ± 3.56^b^	149.89 ± 8.30^a^
Malonylglycitin	ND	78.37 ± 3.77^a^	1,943.29 ± 6.38^b^	2,374.03 ± 9.24^a^
Malonylgenistin	1,141.39 ± 68.62^a^	887.43 ± 27.64^b^	ND	ND
Daidzein	ND	ND	747.73 ± 7.52^b^	908.80 ± 29.10^a^
Glycitein	ND	ND	ND	64.78 ± 2.77^a^
Genistein	ND	ND	ND	ND
Total	2,994.28 ± 35.89^b^	3,630.05 ± 131.46^a^	3,148.23 ± 0.25^b^	3,832.88 ± 64.77^a^

Effects of UV-B on the content of individual isoflavone in soybean hypocotyl and cotyledon calluses. ND meant not detected. The lower case letters in the same row indicated significant difference at *p* < 0.05 between CK and UV-B in the same callus. *T*-test was used. The data were presented as mean ± SD, *n* = 4.

### 3.4. The key enzyme activity

**FIGURE 3:**
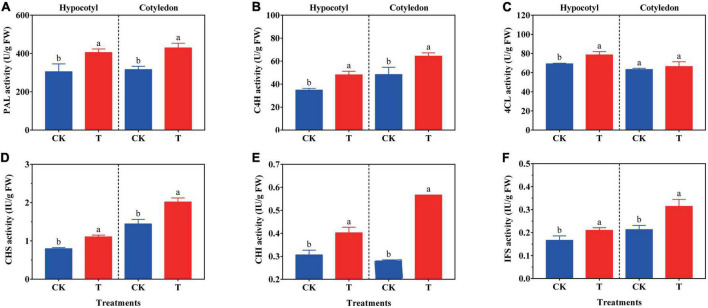
Effects of UV-B on PAL **(A)**, C4H **(B)**, 4CL **(C)**, CHS **(D)**, CHI **(E)**, and IFS **(F)** activity of soybean calluses. The lower case letters indicated significant difference at *p* < 0.05 between treatments, and *T*-test was used for data analysis. The data were presented as mean ± SD, *n* = 3.

The synthesis of isoflavone is related to the key enzyme activity and its gene expression level in the synthetic metabolic pathway. The activity of PAL, C4H, 4CL, CHS, CHI, and IFS in soybean calluses was investigated (Figure 3). In soybean hypocotyl callus, the activity of PAL, C4H, 4CL, CHS, CHI, and IFS increased by 14.08, 38.07, 13.53, 38.75, 31.37, and 26.08% compared with the control, respectively. And the activity of PAL, C4H, CHS, CHI, and IFS significantly increased by 35.83, 33.13, 40.28, 101.14, and 47.42% in soybean cotyledon calluses under UV-B radiation, respectively, compared with the control. But there was no difference on the 4CL activity between the treatment and control group.

### 3.5. The gene expression of key enzymes

**FIGURE 4:**
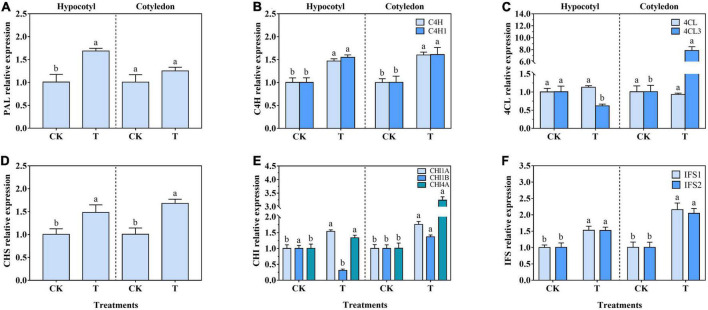
Effects of UV-B on PAL **(A)**, C4H **(B)**, 4CL **(C)**, CHS **(D)**, CHI **(E)**, and IFS **(F)** relative expression of soybean calluses. The lower case letters indicated significant difference at *p* < 0.05 between treatments, and *T*-test was used. The data were presented as mean ± SD, *n* ≥ 3.

The relative expression of *PAL*, *C4H*, *4CL*, *CHS*, *CHI*, and *IFS* in soybean calluses was generally consistent with their activity (Figure 4). The relative expression of *PAL*, *C4H*, *C4H1*, *CHS*, *CHI1A*, *CHI4A*, *IFS1*, and *IFS2* increased by 67.33, 47.00, 55.00, 46.54, 53.39, 32.67, 52.38, and 50.50% in soybean hypocotyl callus under UV-B radiation, respectively. There was no significant difference on *4CL* expression in soybean hypocotyl callus (Figure 4C). Interestingly, *4CL3* and *CHI1B* expression significantly reduced by 38.61 and 69.00%, respectively, compared with the control (Figures 4C, E). Besides, the relative expression of *C4H*, *C4H1*, *4CL3*, *CHS*, *CHI1A*, *CHI1B*, *CHI4A*, *IFS1*, and *IFS2* increased by 60.00, 59.41, 679.21, 66.34, 74.26, 36.45, 220.79, 113.86, and 101.98% in soybean cotyledon callus under UV-B radiation, respectively. And there was no significant difference on *PAL* and *4CL* expression.

## 4. Discussion

Ultraviolet B radiation caused oxidative damages of cells and affected the synthesis of various bioactive metabolites in soybean calluses (Figures 1, 2). Total flavonoids content increased by 13.84 and 76.54% in soybean hypocotyl and cotyledon calluses under UV-B radiation, respectively, as well as their antioxidant activity (Figure 2), indicating that the accumulation of flavonoids is the result of plant stress resistance (16). UV-B significant effects on some physiological indicators, antioxidant activity, and endogenous hormone levels during the growth and development of plants (17). Alvero-Bascos and Ungson (18) revealed that the level of three flavonoids (apigenin, vitexin, and isovitexin) was 20-fold higher than the control in callus cultures of jatropha (*Jatropha curcas* L.) exposed to UV-B (12.6 and 25.3 kJ/m^2^). Besides, many phenolic compounds were synthesized *in vitro*-grown plant materials in *Echium orientale* L under UV-B radiation, especially rosmarinic acid and quercetin (7), and their concentrations were highly correlated with the antioxidant activity (19). Applying ultraviolet radiation has been a simple and effective technology to biofortify plant tissue with secondary metabolites (20). Hence, UV-B radiation was necessary for the synthesis of flavonoids and some other plant secondary metabolites in plant cells and tissues.

Ultraviolet B radiation (40 μW/cm^2^) induced the synthesis of isoflavones, and their content increased significantly by 21.23 and 21.75% in soybean hypocotyl and cotyledon calluses, respectively, (Table 1). Previous study has shown that total isoflavone content is about 8,000 μg/g in 4-day-old soybean sprout under UV-B treatment (3), but it is up to half of the entire soybean sprout in each callus. It might due to the stress resistance capacity of plants varies between different development stages under UV-B radiation (21). Plants are more sensitive to UV-B during the early stage of development. Because the epidermal cell wall is thin and the stability of genes is low. Also, the effects are related to UV-B radiation intensity and radiation time (22). Therefore, the combination of tissue culture technique and UV-B radiation treatment is an effective way to enrich biological metabolites, such as isoflavone. In addition, callus culture technique cannot be affected by the external environment, with the advantages of conditional controlling, stable process, and resource consumption reduction.

The content of total isoflavone was basically same in soybean hypocotyl and cotyledon calluses under UV-B irradiation. However, the composition and content of individual isoflavone was different (Table 1; Supplementary Figure 2). Four individual isoflavones content was higher in soybean hypocotyl callus, including daidzin, genistin, malonyldaidzin and malonylglycitin. Malonylglycosides were the main isoflavone. Interestingly, glycitin, genistin, malonyldaidzin, malonylglycitin, and daidzein was detected in soybean cotyledon callus (Supplementary Figure 2). Compared with isoflavone in soybean hypocotyl callus, the types of isoflavone was more abundant in soybean cotyledon callus, and their content increased significantly under UV-B radiation (Table 1). The difference in isoflavone composition and content of soybean callus might be resulted from the specific sensitivity of different tissues to UV-B irradiation (23). The biosynthesis of isoflavone is also related to the degree of differentiation, soybean varieties and environmental conditions (24). In addition, soybean callus, with well growth and division ability, is a kind of undifferentiated parenchymatous cells. The function of individual isoflavone is different in soybean callus. Glycosidic isoflavone, which is mainly used to form cell parenchyma or organelle membrane to maintain the structural stability of cells, is abundant in callus (1). UV-B radiation can accelerate the conversion between malonyl-glycosidic and glycosidic isoflavone (25). Daidzein and genistein, which could induce nod gene to activate nodulation process (26), were related to the regulation of bacterial communities in soybean root, so their contents were lower in soybean hypocotyl and cotyledon calluses (Table 1). Hence, the response of soybean hypocotyl and cotyledon calluses to UV-B radiation was different. According to their characteristics and specific sensitivity to UV-B, soybean callus can be used to enrich specific isoflavone as needed.

The composition and content of isoflavone was related to the regulation of activities and gene expression levels of PAL, C4H, 4CL, CHS, CHI, and IFS in isoflavone biosynthetic pathway (Figures 3, 4). Isoflavones could be accumulated by positively regulating the activity of key enzymes in soybean hypocotyl and cotyledon calluses exposed to UV-B compared with the control (Figure 3), especially CHS, CHI and IFS activity. The relative expression of key enzymes was basically consistent with their activity (Figure 4). PAL is the initial enzyme of phenylpropane metabolic pathway, including ten genes encoding for PAL found in the *L. japonicus* genome (27). All the genes encoding for enzymes of isoflavone pathway can be strongly induced under UV-B radiation, especially *LjPAL1*. Reports have shown that the transcription levels of *C4H*, *4CL*, and *IFS1* also increased significantly in soybean induced by UV-B stress, promoting the accumulation of polyphenols including *p*-coumaric acid, cinnamic acid and isoflavone (27). Besides, CHS, which connected phenylpropane metabolic pathway and isoflavone biosynthetic pathway, plays a vital role in the synthesis of isoflavone biosynthetic precursor molecules (naringenin chalcone and isoliquiritigenin) (28). UV-B radiation could stimulate the expression of *GmCHS1*, *GmCHS3*, *GmCHS4*, *GmCHS6*, and *GmCHS7* in soybean hypocotyl, improve the synthesis rate of naringenin chalcone and isoliquiritigenin, and promote the accumulation of daidzein, genistein and other isoflavone monomers (29). The activity and expression of CHS was up-regulated in soybean hypocotyl and cotyledon calluses under UV-B radiation (Figures 3D, 4D), promoting the transformation of 4-coumarinyl-CoA into isoflavone synthesis pathway. *IFS1* and *IFS2* plays a catalytic role in isoflavone synthesis, and *IFS2* is more likely to be adjusted by UV-B than *IFS1* (29). *IFS2* expression increased significantly in soybean under UV-B radiation, promoting the synthesis of genistein and daidzein, and improving the defense ability of soybean (30). The contents of daidzein and genistein in callus culture of *Pueraria lobata* were also closely related to the activity of IFS for isoflavone synthesis (31). Interestingly, results showed that *IF2* expression up-regulated in both hypocotyl and cotyledon calluses, but only daidzein was higher in content in soybean cotyledon callus, while genistein was not detected (Figure 4F; Table 1). It might due to the difference in the composition of endogenous isoflavones among soybean varieties (24). However, the expression of *4CL3* and *CHI1B* in soybean hypocotyl callus under UV-B radiation decreased significantly compared with the control (Figures 4C, E), because there might be a time lag between gene expression of key enzymes and isoflavone synthesis (32). The transcription and translation of key enzymes also affected the accumulation of bioactive metabolites. In addition, the sensitivity of this process to light stress was different. In sum, UV-B radiation was effective to enrich isoflavone in soybean calluses by up-regulating the gene expression and activity of key enzymes.

## 5. Conclusion

Isoflavones accumulated with specificity in soybean hypocotyl and cotyledon calluses under UV-B radiation. UV-B radiation promoted isoflavones synthesis with different effects on individual isoflavone, and enhanced antioxidant capacity of soybean calluses by regulating key enzyme (PAL, C4H, 4CL, CHS, CHI, and IFS) gene expression levels and their activity. The main isoflavones in hypocotyl callus were glycosides and malonylglycosides. The isoflavones in cotyledon calluses were mainly malonylglycosides and aglycones. These findings provide a new method for enriching specific individual isoflavone and improving the nutritional value of soybean processing products, and will help reduce resource occupation, improve the stability of products, and achieve sustainable development.

## Data availability statement

The original contributions presented in this study are included in the article/Supplementary material, further inquiries can be directed to the corresponding author.

## Author contributions

MW: validation, data curation, formal analysis, and writing—original draft. GL: methodology and data curation. TG: validation and formal analysis. CX: validation, software, and data curation. PW: software and data curation. RY: visualization and writing—review and editing. All authors contributed to the article and approved the submitted version.
