# Supplementary material for: UV-B radiation enhances isoflavone accumulation and antioxidant capacity of soybean calluses
Source: Front Nutr. 2023 Mar 30;10:1139698. doi: 10.3389/fnut.2023.1139698 (PMC10097905; doi:10.3389/fnut.2023.1139698)
Supplement: Supplementary file 1 [file Data_Sheet_1.docx]

**Supplementary material for**

**UV-B radiation enhances isoflavone accumulation and antioxidant capacity of soybean calluses**

Mian Wang, Guannan Liu, Tianwei Guo, Chong Xie, Pei Wang, Runqiang Yang*

College of Food Science and Technology, Nanjing Agricultural University, Nanjing, Jiangsu 210095, People's Republic of China

**Running title**: Isoflavone accumulation of soybean calluses

1. **mail:** Mian Wang: [wangmiandd@163.com](mailto:wangmiandd@163.com)

Guannan Liu: 2022208029@stu.njau.edu.cn

Tianwei Guo: 2022208028@stu.njau.edu.cn

Chong Xie: 2020077@njau.edu.cn

Pei Wang: [wangpei@njau.edu.c](mailto:wangpei@njau.edu.c)n

Runqiang Yang: [yangrq@njau.edu.cn](mailto:yangrq@njau.edu.cn)

*** Correspondences:**

Dr. Runqiang Yang

Tel/Fax: + 86-025-84396293

E-mail: [yangrq@njau.edu.cn](mailto:yangrq@njau.edu.cn)

Address: College of Food Science & Technology, Nanjing Agricultural University, Nanjing, Jiangsu, 210095, People's Republic of China

***Supplementary Materials***

1. **Supplementary methods**

**1.1 Primers used in this study**

**Table S1** Primers Used for qRT-PCR

| Gene | Primer Name | Primer sequences (5ˊ→3ˊ) | Accession number |
| --- | --- | --- | --- |
| *PAL* | Sense | GCTAAGAAGTTGCATGAGATTGA | NM_001357058.1 |
|  | Ant-sense | TCATTGACAAGCTCAGAGAATTG |  |
| *C4H* | Sense | CGATTTGGCCAAAAAATTCGGTG | NM_001250388.3 |
|  | Ant-sense | CCTTTCCGGTGAAGATGTCG |  |
| *C4H1* | Sense | CGTAGAATTTGGCTCTCGCCC | NM_001371219.1 |
|  | Ant-sense | GCTGAAGCCGCCTTCTGAT |  |
| *4CL1* | Sense | CGGTGAAATTTGCATAAGAGGC | NM_001250821.2 |
|  | Ant-sense | AATCCTTTGTATTTGATCAATTCCT |  |
| *4CL3* | Sense | ATCTCCAACCACCTCCCT | NM_001250341.2 |
|  | Ant-sense | GGATTCCGAGGTTGGACAAT |  |
| *CHS* | Sense | GCTATTGATGGACACCTTCG | NM_001371381.1 |
|  | Ant-sense | ACCAGGGTGTGCAATCCA |  |
| *CHI1A* | Sense | CATTGGATGGTCGTGAATACGT | NM_001248290.2 |
|  | Ant-sense | TTGTAGAAAACAGTGGAGCCTG |  |
| *CHI1B* | Sense | CATTGGATGGTCGTGAATACGT | NM_001249826.2 |
|  | Ant-sense | TTGTAGAAAACAGTGGAGCCTG |  |
| *CHI4A* | Sense | ATCTTTGCTTGGCCATGGAAT | NM_001249853.2 |
|  | Ant-sense | AGCTGCCAACCTATCCCT |  |
| *IFS1* | Sense | GAGAGCTGGCCTCACAGTTC | NM_001249093.2 |
|  | Ant-sense | TGCGATGGCAAGACACTACT |  |
| *IFS2* | Sense | TGGAAGTTCGTGAGGAAG | NM_001251586.2 |
|  | Ant-sense | ATGGAGATGGTGCTGTTG |  |
| *EF1b* | Sense | CCACTGCTGAAGAAGATGATGATG | [NM_001249608.2](https://www.ncbi.nlm.nih.gov/nucleotide/NM_001249608.2?report=genbank&log$=nucltop&blast_rank=5&RID=BXWAJE6401R) |
|  | Ant-sense | AAGGACAGAAGACTTGCCACTC |  |

**2. Supplementary Figures and Table**

**2.1 Optimization of culture media**

The configuration of culture media was based on the methods proposed by Ganapathi et al.(1)and Rani et al.(2). It is not conducive to the formation of soybean callus when the concentration of 2,4-D in the medium is lower than 1 mg/L. In this study, 2,4-D (1, 2, 3, 5 mg/L) and 6-BA (0.5 mg/L) were selected to induce soybean callus. The growth state and total isoflavone content of callus were analyzed to determine the hormone formula of culture media. As shown in Table S2, total isoflavone content reached the maximum in soybean hypocotyl and cotyledon callus cultured on MS media with 3% sucrose, 0.7% agar supplemented with 1 mg/L 2, 4-D and 0.5 mg/L 6-BA (pH 5.8) at 25°C in light (16 h/8 h light/dark) for 28 days. Therefore, MS media with 1 mg/L 2,4-D and 0.5 mg/L 6-BA was selected to induce soybean callus.

**Table S2** Isoflavone contents in soybean callus under different culture media

| Type | Content (µg/g) | | | | |
| --- | --- | --- | --- | --- | --- |
|  | Hypocotyl | | Cotyledon | | |
|  | 1/0.5 | 2/0.5 | 1/0.5 | 3/0.5 | 5/0.5 |
| Daidzin | 178.01±1.81^b^ | 229.61±16.32^a^ | 66.69±2.45^a^ | ND | ND |
| Glycitin | ND | ND | 101.63±0.64^a^ | 16.66±2.61^b^ | 22.19±1.43^b^ |
| Genistin | ND | ND | 109.09±1.37^a^ | ND | ND |
| Malonyldaidzin | 944.26±21.25^a^ | 793.67±45.41^b^ | 127.95±8.23^a^ | 52.51±3.17^b^ | 48.72±0.93^b^ |
| Malonylglycitin | ND | ND | 1265.19±28.75^a^ | 486.00±16.16^b^ | 138.52±5.24^c^ |
| Malonylgenistin | 608.72±18.32^a^ | 462.76±1.08^b^ | ND | 91.83±2.12^a^ | 108.28±12.38^a^ |
| Daidzein | ND | ND | 768.78±46.81^ab^ | 719.63±10.79^b^ | 844.95±17.32^a^ |
| Glycitein | ND | ND | ND | ND | ND |
| Genistein | ND | ND | ND | ND | ND |
| Total | 1730.99±33.42^a^ | 1486.03±43.07^b^ | 2439.33±26.74^a^ | 1366.62±13.26^b^ | 1162.66±23.96^b^ |

**Notes:** The content of individual isoflavone in soybean hypocotyl and cotyledon calluses under different culture media. ND meant not detected. The lower case letters in the same row indicated significant difference at *p* < 0.05 among treatments. T-test was used for data analysis in soybean hypocotyl callus. The one-way ANOVA was used for data analysis in soybean cotyledon callus. The data were presented as mean ± SD, n = 3.

**2.2 UV-B radiation system construction**

In this study, soybean hypocotyl callus was chosen as the experimental subject to optimize UV-B radiation intensity and re-incubation time. As shown in Table S3, total isoflavone content increased at first and then decreased in soybean hypocotyl callus with the increase of UV-B intensity. Total isoflavone content was highest under 40 μW/cm^2^ UV-B radiation, and malonylglycosides were the main individual isoflavone.

**Table S3** Isoflavone contents in soybean hypocotyl callus under different UV-B intensity

| Type \ UV-B (μW/cm^2^) | Content (µg/g) | | | |
| --- | --- | --- | --- | --- |
|  | 0 | 20 | 40 | 80 |
| Daidzin | 178.01±1.81^ab^ | 192.83±17.92^a^ | 182.27±8.70^ab^ | 169.86±3.16^b^ |
| Glycitin | ND | ND | ND | ND |
| Genistin | ND | ND | ND | ND |
| Malonyldaidzin | 944.26±21.25^c^ | 1161.09±24.47^ab^ | 1266.41±38.07^a^ | 1129.63±126.01^b^ |
| Malonylglycitin | ND | ND | ND | ND |
| Malonylgenistin | 608.72±18.32^c^ | 753.07±34.09^b^ | 872.63±10.81^a^ | 817.09±58.46^ab^ |
| Daidzein | ND | ND | ND | ND |
| Glycitein | ND | ND | ND | ND |
| Genistein | ND | ND | ND | ND |
| Total | 1730.99±33.42^c^ | 2106.99±32.88^b^ | 2321.31±46.41^a^ | 2116.58±80.19^b^ |

**Notes:** Effects of UV-B intensity on individual isoflavone in soybean hypocotyl calluses. ND meant not detected. The lower case letters in the same row indicated significant difference at *p* < 0.05 among treatments. The one-way ANOVA was used for data analysis. The data were presented as mean ± SD, n = 3.

As shown in Table S4, total isoflavone content increased at first and then decreased in soybean hypocotyl callus with the extension of re-incubation time after UV-B radiation. Total isoflavone content increased by 35.98% when re-incubation time was 12 h. Hence, soybean calluses were irradiated by 40 μW/cm^2^ UV-B for 2 h, and then re-incubated for 12 h in the dark.

**Table S4** Isoflavonoids contents in soybean hypocotyl callus under different re-incubation time

| Type \ Time (h) | Content (µg/g) | | | | | |
| --- | --- | --- | --- | --- | --- | --- |
|  | 0 | 6 | 12 | 24 | 36 | 48 |
| Daidzin | 140.70±2.89^c^ | 169.71±1.36^c^ | 293.40±20.27^a^ | 227.85±22.47^b^ | 235.10±30.55^b^ | 161.47±17.98^c^ |
| Glycitin | ND | ND | ND | ND | ND | ND |
| Genistin | 70.51±5.32^d^ | 90.22±0.39^bc^ | 120.55±19.11^a^ | 102.31±4.68^ab^ | 103.79±0.63^ab^ | 80.76±2.49^cd^ |
| Malonyldaidzin | 1711.81±58.12^c^ | 1671.38±63.56^c^ | 2251.83±30.26^a^ | 1999.93±98.86^b^ | 2069.50±7.75^ab^ | 1865.80±195.53^bc^ |
| Malonylglycitin | 177.20±3.80^b^ | 168.72±3.51^b^ | 191.78±7.81^a^ | 178.21±4.60^b^ | ND | ND |
| Malonylgenistin | 564.09±10.80^d^ | 623.94±5.46^d^ | 765.51±27.04^c^ | 881.79±51.81^b^ | 1006.79±11.85^a^ | 912.22±35.81^b^ |
| Daidzein | ND | ND | ND | ND | ND | ND |
| Glycitein | ND | ND | ND | ND | ND | ND |
| Genistein | ND | ND | ND | ND | ND | ND |
| Total | 2664.31±41.10^c^ | 2723.96±59.86^c^ | 3623.06±10.12^a^ | 3390.09±15.30^a^ | 3415.19±50.78^a^ | 3020.26±246.82^b^ |

**Notes:** Effects of re-incubation time on individual isoflavone in soybean hypocotyl calluses. ND meant not detected. The lower case letters in the same row indicated significant difference at *p* < 0.05 among treatments. The one-way ANOVA was used for data analysis. The data were presented as mean ± SD, n = 3.

**2.3 Growing status and biomass of soybean calluses under UV-B radiation**

The soybean hypocotyl and cotyledon calluses cultured for 28 days were shown in Figure S1A-B.. The biomass of calluses increased significantly in soybean hypocotyl and cotyledon calluses in 3-18 days, and there was no significant difference in the biomass of calluses during 0-3 days and 18-27 days (Figure S1C-D).


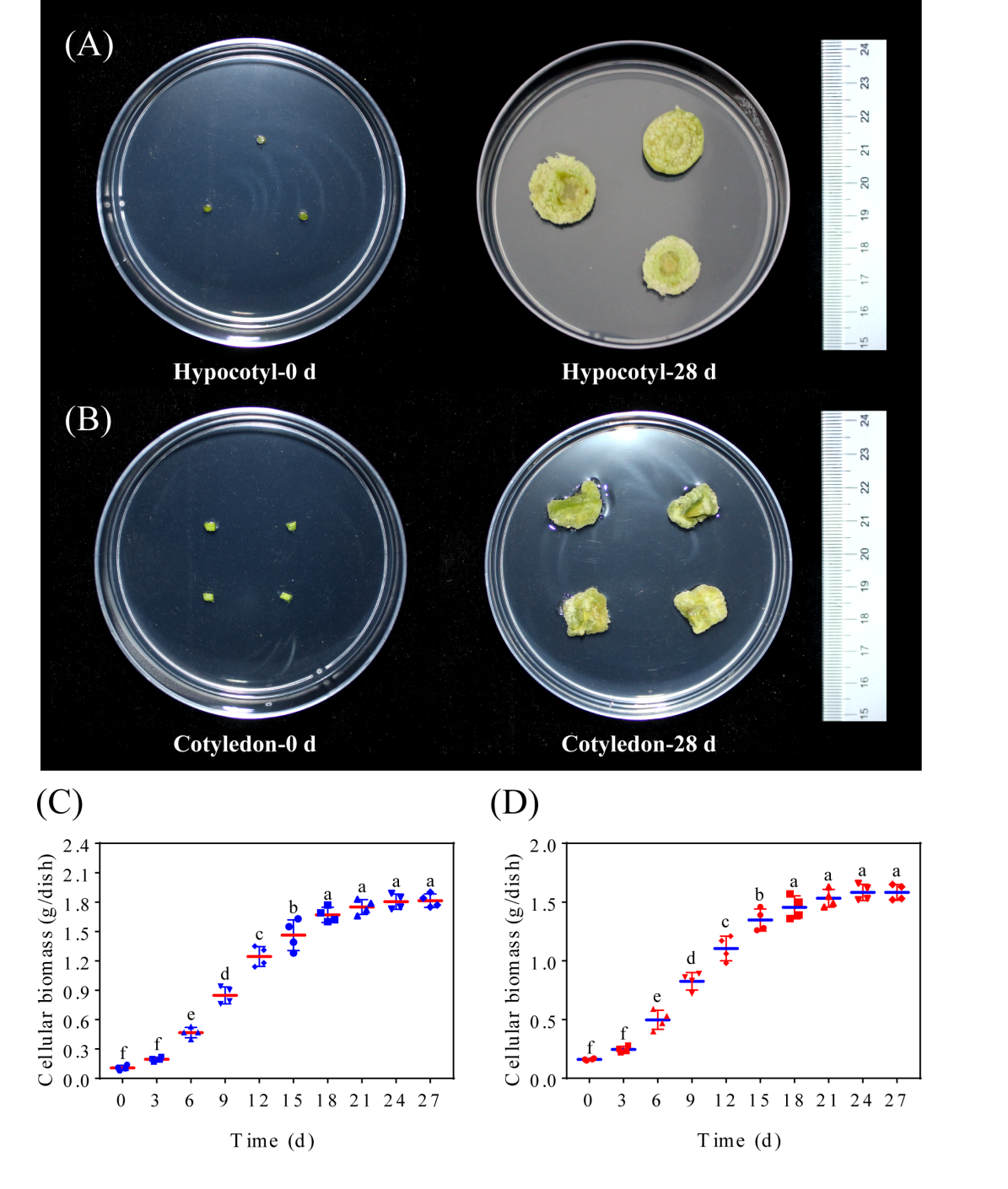


**Figure S1** Effects of UV-B on the growing status and biomass of soybean hypocotyl (A, C) and cotyledon (B, D) calluses. The lower case letters indicated significant difference at *p* < 0.05, and the one-way ANOVA was used for data analysis. The data were presented as mean ± SD, n = 3.

**2.4 Typical HPLC chromatograms of nine isoflavone monomers in soybean hypocotyl and cotyledon calluses**

Typical HPLC chromatograms of nine isoflavone monomers were shown in Figure S2. Nine major isoflavones were detected (Figure S2A). As shown in Figure S2B, daidzin, genistin, malonyldaidzin and malonylgenistin were the main isoflavones in soybean hypocotyl callus. Interestingly, glycitin, genistin, malonyldaidzin, malonylglycitin and daidzein were the main isoflavones in soybean cotyledon callus (Figure S2C).


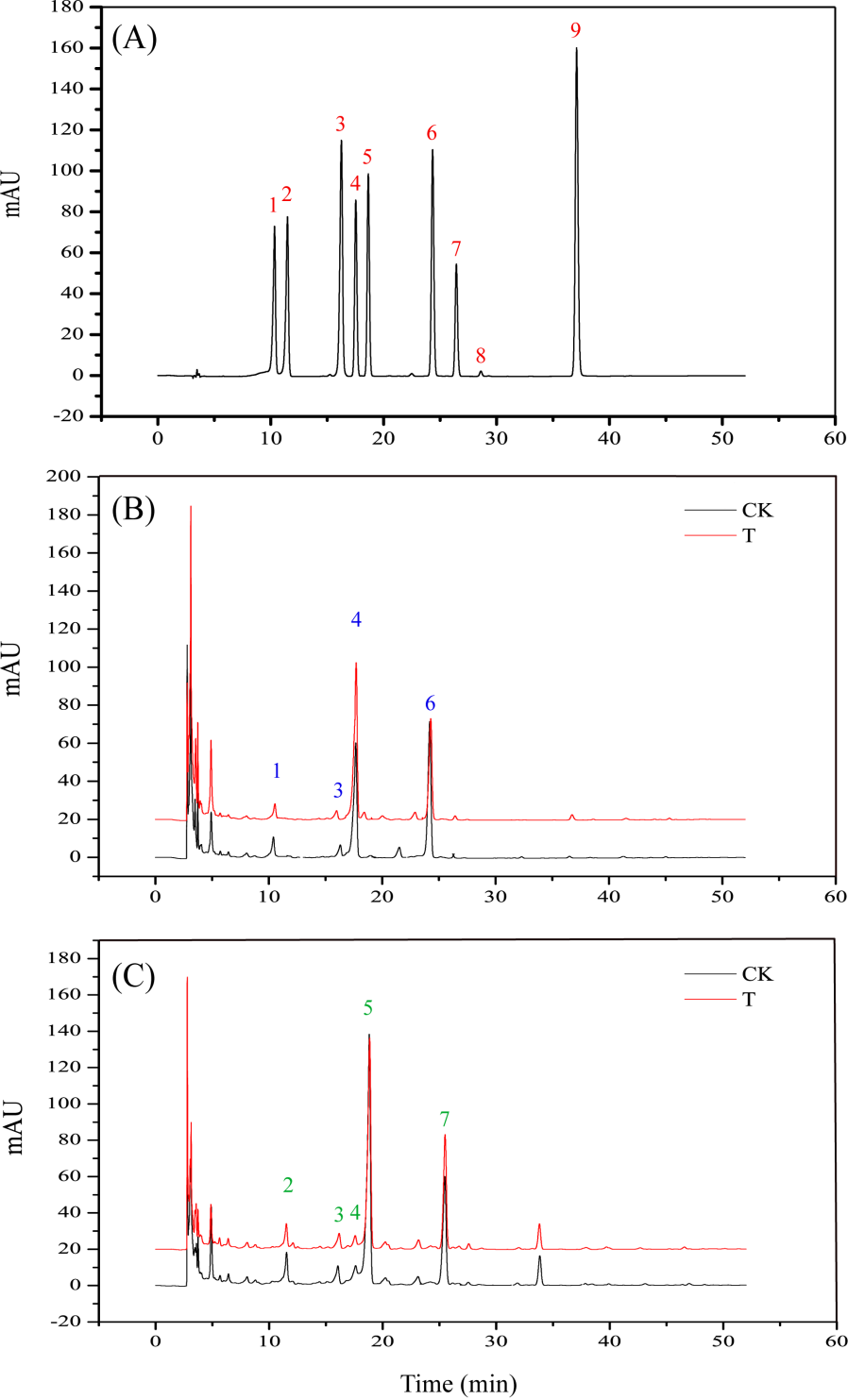


**Figure S2** Typical HPLC chromatograms of nine isoflavone monomers in soybean hypocotyl and cotyledon calluses. A: Typical HPLC chromatograms of nine isoflavone monomers, 1 Daidzin, 2 Glycitin, 3 Genistin, 4 Malonyldaidzin, 5 Malonylglycitin, 6 Malonylgenistin, 7 Daidzein, 8 Glycitein, 9 Genistein; B: Typical HPLC chromatograms of isoflavone in soybean hypocotyl calluses; C: Typical HPLC chromatograms of isoflavone in soybean cotyledon calluses.

**Reference:**

1. Ganapathi TR, Kumar GBS, Srinivas L, Revathi CJ, Bapat VA. Analysis of the limitations of hepatitis B surface antigen expression in soybean cell suspension cultures. *Plant cell Rep*. (2007) 26:1575-84. doi:10.1007/s00299-007-0379-7

2. Rani D, Vimolmangkang S. Trends in the biotechnological production of isoflavonoids in plant cell suspension cultures. *Phytochem Rev*. (2022) 21:1843-62. doi:10.1007/s11101-022-09811-6
